# Functional Identification and Evolutionary Analysis of Two Novel Plasmids Mediating Quinolone Resistance in *Proteus vulgaris*

**DOI:** 10.3390/microorganisms8071074

**Published:** 2020-07-18

**Authors:** Hongyang Zhang, Mingding Chang, Xiaochen Zhang, Peiyan Cai, Yixin Dai, Tongzhen Song, Zhenzhou Wu, Haijin Xu, Mingqiang Qiao

**Affiliations:** 1 The Key Laboratory of Molecular Microbiology and Technology, Ministry of Education, College of Life Sciences, Nankai University, Tianjin 300071, China; zhanghongyang0926@163.com (H.Z.); MacraiseZ@163.com (X.Z.); pycai@connect.hku.hk (P.C.); daiyixin@mail.nankai.edu.cn (Y.D.); tongzhensong16@163.com (T.S.); naturepower@nankai.edu.cn (Z.W.); xuhaijin@aliyun.com (H.X.); 2 Zhengzhou University Industrial Technology Institute Co. Ltd., Zhengzhou 450000, China; chang7653@163.com

**Keywords:** quinolone resistance, *Proteus vulgaris*, *qnrD*-carrying plasmids, *qnrD* expression, direct repeats, homologous recombination

## Abstract

Plasmid-mediated quinolone resistance (PMQR) remains one of the main mechanisms of bacterial quinolone resistance and plays an important role in the transmission of antibiotic resistance genes (ARGs). In this study, two novel plasmids, p3M-2A and p3M-2B, which mediate quinolone resistance in *Proteus vulgaris* strain 3M (P3M) were identified. Of these, only p3M-2B appeared to be a *qnrD*-carrying plasmid. Both p3M-2A and p3M-2B could be transferred into *Escherichia coli*, and the latter caused a twofold change in ciprofloxacin resistance, according to the measured minimum inhibitory concentration (MIC). Plasmid curing/complementation and qRT-PCR results showed that p3M-2A can directly regulate the expression of *qnrD* in p3M-2B under treatment with ciprofloxacin, in which process, *ORF1* was found to play an important role. Sequence alignments and phylogenetic analysis revealed the evolutionary relationships of all reported *qnrD*-carrying plasmids and showed that *ORF1–4* in p3M-2B is the most conserved backbone for the normal function of *qnrD*-carrying plasmids. The identified direct repeats (DR) suggested that, from an evolutionary perspective, p3M-2B may have originated from the 2683-bp *qnrD*-carrying plasmid and may increase the possibility of plasmid recombination and then of *qnrD* transfer. To the best of our knowledge, this is the first identification of a novel *qnrD*-carrying plasmid isolated from a *P. vulgaris* strain of shrimp origin and a plasmid that plays a regulatory role in *qnrD* expression. This study also sheds new light on plasmid evolution and on the mechanism of horizontal transfer of ARGs encoded by plasmids.

## 1. Introduction

The unregulated use of antibiotics has led to the emergence of resistant bacteria carrying different types of antibiotic resistance genes (ARGs), which spread in people, animals, and the environment (water, soil, air, etc.) by horizontal gene transfer (HGT), raising a health challenge [1,2,3,4]. Quinolones (ciprofloxacin, norfloxacin, ofloxacin, etc.) are among the most commonly prescribed antibiotics due to their broad-spectrum antibacterial activity, and the frequent use of quinolones has contributed to the emergence of quinolone resistance worldwide, posing a serious threat to public health [5,6,7,8]. The mechanisms of bacterial quinolone resistance are well understood. For instance, bacteria develop quinolone resistance mainly as a consequence of mutations of proteins such as topoisomerase IV and DNA gyrase [9,10,11]. Recent studies have shown that bacteria, especially clinically important bacteria, gain increased resistance to quinolones by the acquisition of plasmids harboring quinolone resistance genes [12,13,14]. Resistance plasmids play a crucial role in the horizontal transfer of quinolone resistance genes due to their good self-replication and transmission characteristics [15,16]. *qnrD*, a plasmid-mediated quinolone resistance (PMQR) determinant that encodes a pentapeptide repeat protein, induces low susceptibility to quinolone by binding to DNA–DNA gyrase complexes [17,18,19,20]. Unlike other *qnr* alleles (*qnrA*, *qnrB*, *qnrC*, *qnrE*, *qnrS*), *qnrD* is usually harbored by small, non-conjugative plasmids, is closely related to *qnrB* variants [17], and does not produce significant quinolone resistance by itself [18,21,22]. However, it is undeniable that *qnrD* is of vital importance in the transmission and development of quinolone resistance [19,23].

DNA recombination mediated by direct repeats (DR), a typical type of DNA repetition in which two copies of identical or highly similar sequences are arranged in the same orientation along a DNA strand, has been increasingly reported not only in prokaryotes but also in eukaryotes, making it clear that it may be a common genetic and evolutionary mechanism [24,25,26]. Several studies have shown that recombination may occur between DR regions in the chromosome or plasmids of *Escherichia coli*, further manifesting that the existence of DR might increase the possibility of the formation of various products of recombination [26,27,28,29]. Therefore, the role of DR-mediated recombination in the transmission of ARGs should not be neglected.

In this study, we identified two novel plasmids closely associated with enhanced quinolone resistance in a *Proteus vulgaris* strain of shrimp origin and preliminarily describe the possible formation process of *qnrD*-carrying plasmids of specific types via DR-mediated recombination, which might be a widespread and conserved mechanism of *qnrD* transfer from an evolutionary perspective.

## 2. Materials and Methods

### 2.1. Bacterial Strains and Growth Condition

The strains and plasmids used in this study are shown in Table 1. p3M-2A and p3M-2B plasmids were identified from *P. vulgaris* strain 3M (P3M), which was isolated from the intestines of shrimps in Tianjin, China. All strains were cultured in Luria–Bertani (LB) medium at 37 °C.

### 2.2. Plasmid Isolation and Sequencing

The plasmids p3M-2A and p3M-2B harbored by P3M were extracted using TIANprep Mini Plasmid Kit (DP103-03, TianGen, Beijing, China) and then purified with SPARKeasy Gel DNA Extraction Kit (AE0101-C, SparkJade, Qingdao, China). Six restriction endonucleases (*Hin*d III, *Bam*H I, *Sma* I, *Xba* I, *Sac* I, *Sph* I according to the multiple cloning sites of pUC19, Takara, Dalian, China) were used to digest these two plasmids at 37 °C for 1 h; the enzymes with only one restriction site on the plasmids were selected, which were *Hin*d III for p3M-2B and *Sac* I for p3M-2A. The digested plasmid fragments and the pUC19 vector were ligated with T4 DNA ligase (Takara, Dalian, China) according to the manufacturer’s instructions. The ligation products were then transformed into *E. coli* DH5α competent cells (β-galactosidase-deficient strains), and blue colonies were selected. The correct colonies were cultured to extract the plasmids, which were sequenced later by Invitrogen, Beijing, China.

### 2.3. Construction of p3M-2A* and p3M-2B*

p3M-2A lacking *ORF1* (p3M-2A*) and p3M-2B lacking *ORF5* (p3M-2B*) were generated to verify the function of *ORF1* and *ORF5*, respectively. Oligonucleotide primers with restriction sites were designed to generate linear fragments of p3M-2A and p3M-2B (Table S1). Amplified fragments were digested with the endonuclease *Bam*HI and ligated with T4 DNA ligase to form the circular plasmids p3M-2A* and p3M-2B*.

### 2.4. Plasmid Transformation

The plasmids were transformed into *E. coli* DH5α competent cells by chemical transformation to yield different DH5α derivatives. For P3M derived strains, the plasmids were first transferred into *E. coli* S17-1 to form intermediate transformants and then transferred into the recipient strain by the parental conjugation method [31]. Transformants were selected on LB agar plates by colony PCR, using specific primers (Table S1).

### 2.5. Antimicrobial Susceptibility Testing

The MIC of both wild-type (WT) strains and transformants were determined by the broth microdilution method according to the Clinical & Laboratory Standards Institute (CLSI) recommendations [32].

### 2.6. Spot Growth Assays

*P. vulgaris* and *E. coli* strains were cultured overnight in LB medium at 37 °C. The cultures were transferred into fresh LB medium and grown up to OD_600_ of 0.6. After gradient dilution, 5 μL droplets of the cultures were dropped onto LB agar plates containing 0.25 mg/L or 0.05 mg/L ciprofloxacin, and the plates were incubated upside down at 37 °C for 24 h [33].

### 2.7. Phylogenetic Analysis and Sequence Alignment

Unrooted neighbor-joining trees of the *qnrD*-carrying plasmids were generated from the indicative aligned sequences using MEGA7 (Temple University, Philadelphia, Pennsylvania, USA) [34]. Linear comparison and map generation of the plasmids were performed and visualized using Easyfig 2.2.3 software (University of Queensland, Brisbane, Australia) [35].

### 2.8. Plasmid Curing

Overnight cultured P3M was transferred to fresh LB medium containing 0.25% of sodium dodecyl sulfonate (SDS) and cultured at 37 °C until reaching the late log phase. After that, the cultures were transferred again and cultured according to the method described by Verma et al. [36]. The cultures were diluted and transferred onto LB agar plates without antibiotics after several repetitions of SDS treatments and culturing, and the plasmid-deleted strains were screened by colony PCR using specific primers (Table S1).

### 2.9. Quantitative Real-Time PCR

Total bacterial RNA was isolated using an RNAprep Pure Cell/Bacteria Kit (DP430, TianGen, Beijing, China) and then reverse-transcribed into cDNA using the PrimeScript™ RT reagent Kit (RR047A, Takara, Dalian, China) according to the manufacturer’s instructions. The cDNAs of each sample were diluted to 100 ng/μL as templates for qRT-PCR experiments, which were repeated three times independently (three biological replicates). Specific primers for *qnrD* were designed based on the plasmid sequence of p3M-2B (Table S1). We used 16S rDNA as the endogenous reference gene to normalize the expression of the target genes in each cDNA template. The relative mRNA concentration was calculated by the comparative threshold cycle (2^−∆∆CT^) method.

### 2.10. Accession Number

The complete sequences of plasmids p3M-2A and p3M-2B were deposited in GenBank, with accession numbers JX514065 and JX514066, respectively.

## 3. Results

### 3.1. Effect of p3M-2A on the Expression of qnrD in p3M-2B

**Figure 1:**
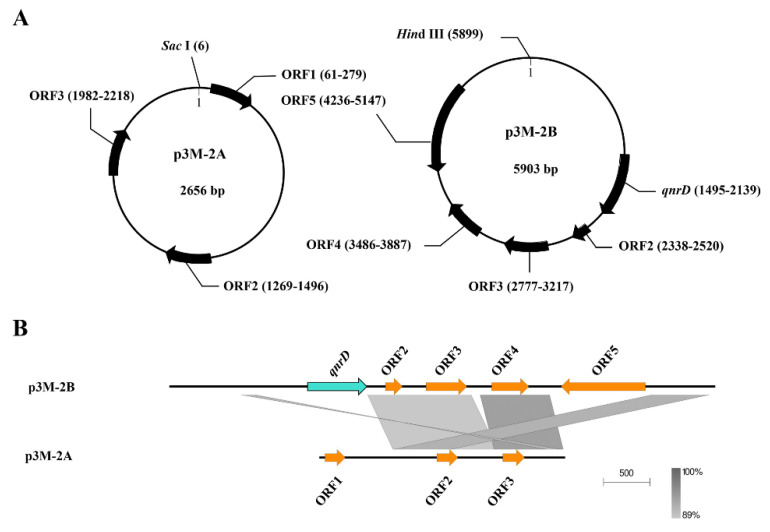
Graphical maps (**A**) and comparison of the structures (**B**) of p3M-2A and p3M-2B. The grey and dark shading in (b) indicates common regions between the plasmids.

The plasmids p3M-2A and p3M-2B of P3M were sequenced, and their total length resulted to be 2656 bp and 5903 bp, respectively (Figure 1A). No resistance genes were present in p3M-2A, while p3M-2B carried *qnrD*. In this study, we found a certain correlation between these two plasmids—a large portion of the entire p3M-2A sequence shared a relatively high sequence identity (>89%) with *ORF2*–*ORF4* and their intergenic regions of p3M-2B, implying that these two plasmids may originate from the same plasmid backbone (Figure 1B). Besides, we did not find any sequence with high identity with *ORF1* of p3M-2A in the database, and the same was observed for *ORF5* of p3M-2B.

In order to verify the function of p3M-2A and p3M-2B, different plasmid deletion strains of P3M were generated: P3M-∆2A (p3M-2A elimination), P3M-∆2B (p3M-2B elimination), and P3M-∆2A2B (p3M-2A and p3M-2B elimination). In addition, p3M-2A and p3M-2B were transferred into *E. coli* DH5α separately or simultaneously to generate transformants with different plasmids: *E. coli* DH5α-2A (transformant containing p3M-2A), *E. coli* DH5α-2B (transformant containing p3M-2B), and *E. coli* DH5α-2A2B (transformant containing p3M-2A and p3M-2B). The ciprofloxacin MIC of these strains were determined. As shown in Table 2, ciprofloxacin resistance was decreased in P3M-∆2A and P3M-∆2B compared with P3M. Likewise, the resistance to ciprofloxacin was also reduced in *E. coli* DH5αtransformants containing only p3M-2A or p3M-2B compared with *E. coli* DH5α-2A2B. These results showed that the presence of both plasmids caused an eight- or four-fold change in the ciprofloxacin resistance of P3M and *E. coli* DH5α-2A2B compared with the plasmid-free strains, respectively. In addition, we found that the ciprofloxacin resistance of P3M-∆2A and *E. coli* DH5α-2B strains containing only p3M-2B increased by four times or two times, respectively, while p3M-2A did not contribute to strain resistance. These results indicated that the two plasmids played synergistic roles in improving strain resistance to ciprofloxacin, and p3M-2B had a more obvious promoting effect. It is worth noting that, despite the absence of *qnrD*, p3M-2A still played a specific role in improving the quinolone resistance of the bacteria tested.

**Figure 2:**
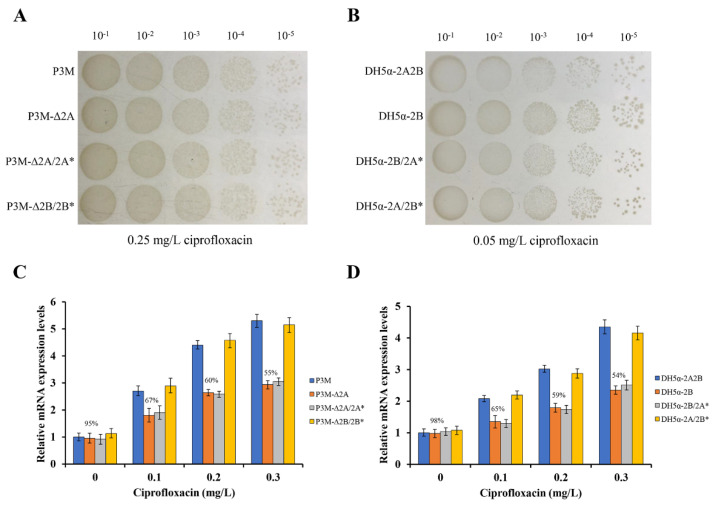
(**A**) Spot growth assays of wild-type (WT) P3M, p3M-2A deleted strain (P3M-∆2A), p3M-2A* complemented strain (P3M-∆2A/2A*), and p3M-2B* complemented strain (P3M-∆2B/2B*) on LB agar in the presence of 0.25 mg/L of ciprofloxacin. (**B**) Spot growth assays of DH5α transformant containing both plasmids (DH5α-2A2B), DH5α transformant containing p3M-2B (DH5α-2B), DH5α-2B strain complemented with p3M-2A* (DH5α-2B/2A*), and DH5α-2A strain complemented with p3M-2B* (DH5α-2A/2B*) on LB agar in the presence of 0.05 mg/L of ciprofloxacin. (**C**) Expression of *qnrD* in P3M, P3M-∆2A, P3M-∆2A/2A*, and P3M-∆2B/2B* with different ciprofloxacin concentrations. (**D**) Expression of *qnrD* in DH5α-2A2B, DH5α-2B, DH5α-2B/2A*, and DH5α-2A/2B* with different ciprofloxacin concentrations.

To confirm the function of p3M-2A, we carried out spot growth assays to test the effect of the absence of p3M-2A on the stability of p3M-2B. After subcultured for 100 generations, P3M-∆2A and DH5α-2B were able to preserve the ciprofloxacin-resistant phenotype in the presence of 0.25 mg/L and 0.05 mg/L of ciprofloxacin, respectively (Figure 2A,B), showing that p3M-2B could replicate and function independently and the absence of p3M-2A had no effect on the stability of p3M-2B in either P3M or *E. coli* DH5α strains. Considering that p3M-2A could improve the ciprofloxacin resistance of the strain even in the absence of *qnrD*, we speculated that this plasmid may have specific regulatory functions. Thus, we measured the expression level changes of *qnrD* in the presence or absence of p3M-2A using qRT-PCR (Figure 2C,D). The expression level of *qnrD* in P3M and P3M-∆2A showed little difference in the absence of ciprofloxacin (Figure 2C). As the concentration of ciprofloxacin increased, the expression level of *qnrD* in both strains increased to different degrees; *qnrD* expression in P3M-∆2A was apparently lower than that in the wild-type strain, and the difference became more obvious as the concentration increased. Similarly, the expression of *qnrD* showed the same changing trend in *E. coli* DH5α transformants containing p3M-2B and p3M-2A2B (Figure 2D). These results indicated that p3M-2A played a positive regulatory role in the expression of *qnrD* in p3M-2B, that is, the higher the ciprofloxacin concentration in the environment, the more obvious the regulatory effect of p3M-2A.

Since the sequence of *ORF2*–*ORF3* in p3M-2A shares relatively high identity with that of p3M-2B, while *ORF1* does not (Figure 1B), we speculated that *ORF1* might play a certain regulatory role in *qnrD* expression. Hence, we constructed a new p3M-2A plasmid deleting *ORF1* (p3M-2A*) and transferred it into P3M-∆2A and DH5α-2B to verify the effect of *ORF1*. As shown in Figure 2A and 2B, the new plasmid p3M-2A* did not affect the normal replication of plasmid 2B in P3M-∆2A and DH5α-2B, and *qnrD* expression in the transformants was subsequently detected. As expected, p3M-2A* failed to restore the expression of *qnrD* to the wild-type level, but the expression was similar to that of the p3M-2A-deficient strain (Figure 2C,D). These results indicated that *ORF1* in P3M-2A was key to regulate *qnrD* expression, as p3M-2A without *ORF1* could not play its important regulatory function.

### 3.2. Phylogenetic Analysis of qnrD-Carrying Plasmids

By analyzing all the *qnrD*-carrying plasmids reported so far, we found that almost all of them were harbored by bacteria isolated from common environmental “reservoirs” like human and animal intestine, urinary tract, feces, water body, etc. As shown in Table S2, 47 *qnrD*-carrying plasmids have been reported to date, of which most of them were isolated from Enterobacteriaceae, with the genus *Proteus* accounting for a relatively large proportion.

**Figure 3:**
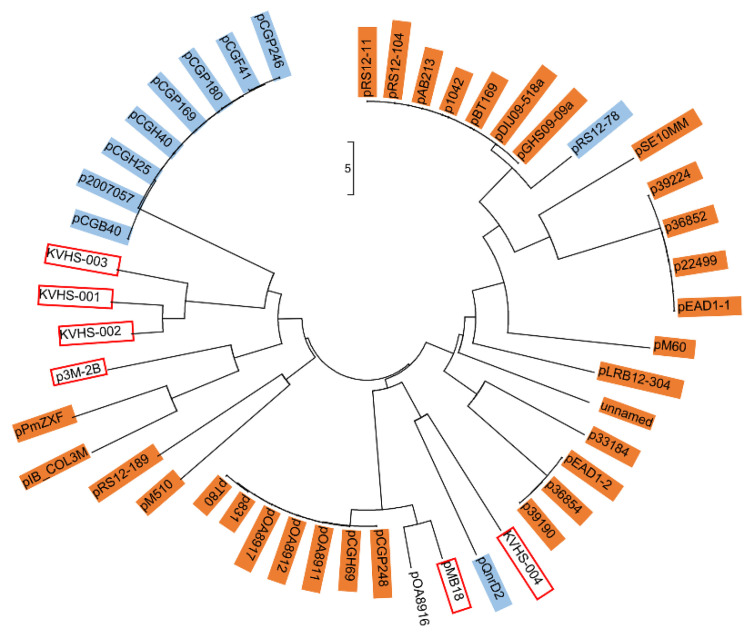
Neighbor-joining phylogenetic tree of p3M-2B with other reported *qnrD*-carrying plasmids. Plasmids of 2.7 kb or 4.3 kb are highlighted in orange or blue, respectively. Plasmids larger than 4.3 kb are marked with red boxes. The evolutionary history was inferred using the neighbor-joining method [38]. The optimal tree with the sum of branch length of 102.98104110 is shown. The tree is drawn to scale, with branch lengths measured in the same units as those of the evolutionary distances used to infer the phylogenetic tree. The evolutionary distances were computed using the Maximum Composite Likelihood method [39] and are expressed as the number of base substitutions per site. The analysis involved 49 nucleotide sequences. The codon positions included were 1st + 2nd + 3rd + Noncoding. All positions containing gaps and missing data were eliminated. There were a total of 645 positions in the final dataset.

Almost all *qnrD*-carrying plasmids reported to date can be roughly divided into 2.7-kb and 4.3-kb categories according to their size [17,18,37]. Interestingly, the p3M-2A plasmid in this study was about 2.7 kb in size, although it did not carry *qnrD*. The size of the p3M-2B carrying *qnrD* was 5.9 kb, and thus it did not comply with the above classification characteristics. In addition, we also found other six special cases that did not conform to the size rule: KVHS-001 (6.9 kb), KVHS-002 (8.1 kb), KVHS-003 (9.4 kb), KVHS-004 (6.2 kb), pOA8916 (2.0 kb), and pMB18 (5.2 kb) (Table S2). In order to further understand the genetic relationship between these *qnrD*-carrying plasmids, a neighbor-joining phylogenetic tree was constructed. As shown in Figure 3, plasmids of the same category tend to be more closely related to each other, while plasmids isolated from *Proteus* were dispersed and could be found in each clade, further illustrating the important function of *Proteus* species as disseminators in the phylogenetic evolution of *qnrD*-carrying plasmids.

### 3.3. Possible Formation Process of p3M-2B

**Figure 4:**
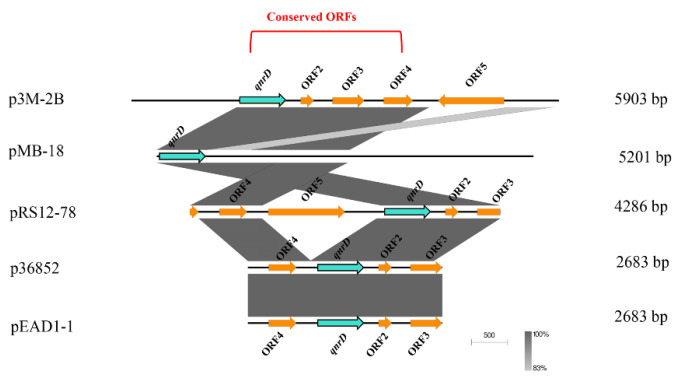
Linear comparison of p3M-2B with other closely related *qnrD*-carrying plasmids isolated from *P. vulgaris* strains.

Due to the relatively large size of p3M-2B compared with the 2.7 and 4.3 kb *qnrD*-carrying plasmids, we hypothesized that it may have a specific formation process. We first focused on the other four plasmids isolated from *P. vulgaris* strains which were different in size and made sequence alignments. As shown in Figure 4, *ORF1* (*qnrD*)-4 of p3M-2B was completely conserved in these four *qnrD*-carrying plasmids, with sequence identity up to 100%. Besides, the 2683-bp plasmids p36852 and pEAD1-1 appeared to be exactly composed of *ORF1–4*, implying that these four conserved genes of p3M-2B are the most conserved genes of the *qnrD*-carrying plasmids, necessary for their normal function.

**Figure 5:**
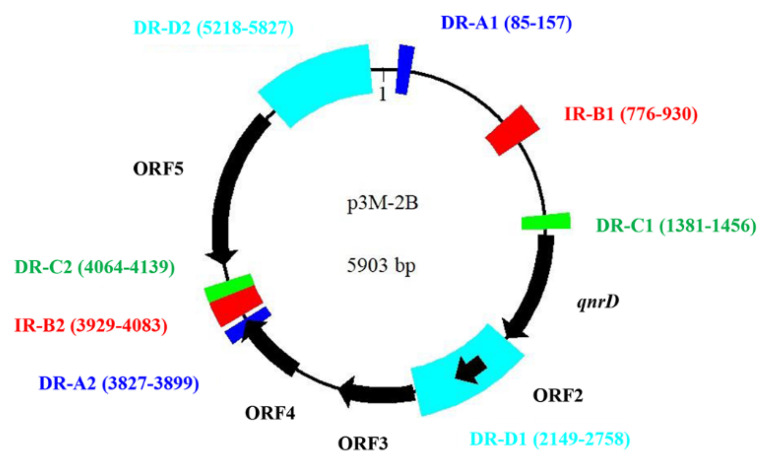
Graphical map of p3M-2B including direct repeats (DR) and inverted repeats (IR). Motifs of the same color represent a pair of DR or IR regions.

Subsequently, the p3M-2B plasmid sequence was analyzed with RepeatAround, a Windows-based software tool designed to find repeats from 3 bp to 64 bp of length in circular genomes [40]. We found that there were four pairs of DR or inverted repeats (IR) in p3M-2B (Figure 5), and it was notable that the sequence length between DR-C1 and -C2 was precisely 2683 bp, which fitted into the category of 2683-bp *qnrD*-carrying plasmids. In particular, the sequence between C1 and C2 corresponded exactly to *ORF1–4*, which appeared to be the most conserved and essential genetic component of *qnrD*-carrying plasmids for their proper function (Figure 4). It has been reported that DNA recombination mediated by DR regions is a major cause of genome plasticity [36,41], so it is conceivable that the DR present in p3M-2B would have a similar function. We hereby supposed that the 5903-bp p3M-2B may be originally derived from a 2683-bp plasmid, and DR-C1 and C2 play an important role in this process.

To test the above hypothesis, a new 2683-bp p3M-2B plasmid without *ORF5* (p3M-2B*) was constructed, and its stability and *qnrD* expression in the transformants P3M-∆2B/2B* and DH5α-2A/2B* (Figure 2) were tested. Just as we expected, p3M-2B* was stable in the transformed strains (Figure 2A,B), and the expression of *qnrD* at different ciprofloxacin concentrations was not affected by *ORF5* deficiency (Figure 2C,D). These results further indicated that *ORF1–4* in *qnrD*-carrying plasmids is a very conserved backbone, and *ORF5* in p3M-2B, which appears to be an acquired exogenous sequence, does not affect the normal replication process and further quinolone resistance,.

**Figure 6:**
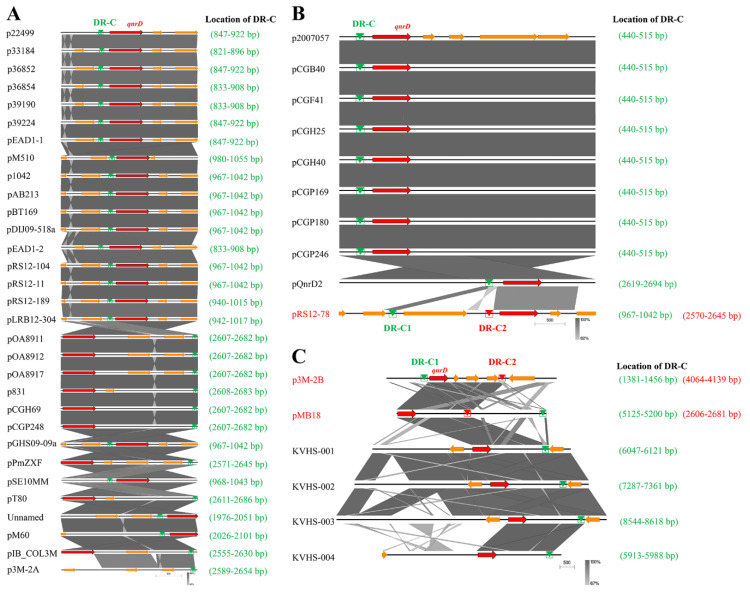
Comparative linear maps of *qnrD*-carrying plasmids of (**A**) 2.7 kb, (**B**) 4.3 kb, and (**C**) larger than 4.3 kb; *qnrD* and other predicted coding sequences (CDS) are denoted by red and orange arrows, respectively. The size of the arrows is to scale. The green and red triangles represent the regions sharing high sequence identity with DR-C in p3M-2B.

To further explore whether this is applicable to all larger *qnrD*-carrying plasmids, we made sequence alignments of the DR-C region in p3M-2B with similar regions in other reported *qnrD*-carrying plasmids. As shown in Figure 6, we found that although all plasmids contained DR regions sharing high sequence identity with DR-C, sequences varied slightly among different plasmids. The sequence with high identity to DR-C was present only in one site in the 2.7-kb plasmids, located upstream of *qnrD* (Figure 6A). It should be noted that although p3M-2A does not carry *qnrD*, it was also included in Figure 6A because it appeared to be in the 2.7-kb plasmid category and share high sequence identity with DR-C. Likewise, all 4.3-kb plasmids contained only one site identical with DR-C, with the exception of plasmid pRS12-78, containing two DR-C regions at different positions (Figure 6B). As shown in Figure 6C, among all plasmids larger than 4.3 kb, only pMB18 appeared to be consistent with p3M-2B, with two different DR regions (DR-C1 and C2), while only one DR-C was found in the four larger *qnrD*-carrying fragments isolated from *Salmonella*, which was unexpected. Plasmid pOA8916, smaller than 2.7 kb, contained one spot of DR-C upstream of *qnrD* as well (not shown). In summary, among all the *qnrD*-carrying plasmids analyzed, only p3M-2B, pMB18, and pRS12-78 presented two different DR regions, and the sequence lengths between them resulted to be exactly 2683 bp, containing *ORF1–4*. Notably, these three plasmids were isolated from *P. vulgaris* strains, which hints at the important host role of *P. vulgaris* in plasmid propagation.

**Figure 7:**
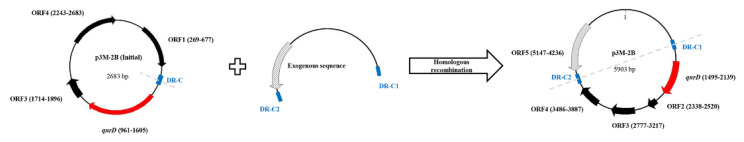
Possible model for in vivo formation of 5903-bp p3M-2B.

Based on the structural characteristics of the above three plasmids, we propose a potential model for the development of larger *qnrD*-carrying plasmids like p3M-2B (Figure 7). The p3M-2B backbone with an initial size of 2.7 kb contains DR-C region about 35–115 bp upstream of *qnrD*. The exogenous sequence containing DR-C at both ends in the adjacent environment may undergo homologous recombination with the DR-C region in p3M-2B (2.7 kb) and thus be integrated to turn into a new *ORF*. Ultimately, a novel plasmid p3M-2B of 5.9 kb is formed.

## 4. Discussion

The *Proteus* genus consists of Gram-negative opportunistic pathogens ubiquitous in the intestine of humans and animals as well as in the natural environment, usually causing clinical infections in patients [42,43,44]. Quinolones are commonly used in the treatment of infections caused by *Proteus*, which, as a result, leads to an increase in the number of quinolone-resistant bacteria. As shown in many well-studied *Proteus* strains, *qnr* alleles are really important in reducing the bacterial sensitivity to quinolones, and, in particular, *qnrD* confers relatively low quinolone resistance and can be transferred via plasmid transformation. The data presented in this article further indicate that *qnrD* is present in small non-conjugative plasmids, and the species characteristics of the host strains of these *qnrD*-carrying plasmids indicate that these plasmids may have first originated from Enterobacteriaceae [19,23] and were then gradually transferred to other bacterial genera through HGT, in which process, *Proteus* spp. presumably played important roles as intermediate hosts. *qnrD*-carrying plasmids were classified in two major types according to their size [17,18,37]. Interestingly, plasmid p3M-2A without *qnrD* in P3M exhibited the size considered by this classification, while p3M-2B did not. Sequence alignment results showed that there was no sequence in the database with high identity with *ORF1* of P3M-2A or *ORF5* of P3M-2B, implying that the two *ORFs* were obtained by p3M-2A and p3M-2B in a specific way from the genome of P3M or unknown exogenous sources. The existence of DR-C in p3M-2A (Figure 6A) and the high sequence identity between p3M-2A and p3M-2B led us to speculate that these two plasmids may share a common ancestor, the 2.7-kb p3M-2B (Figure 7). Subsequently, with the occurrence of homologous recombination in the process of evolution, the *qnrD* gene on some copies of the 2.7-kb p3M-2B was excised, and a completely new sequence (*ORF1*) was obtained to form the p3M-2A plasmid. And as a result, the size of p3M-2A did not change too much, remaining about 2.7 kb. The other copies of the 2.7-kb p3M-2B directly obtained a new sequence (*ORF5*) and finally formed the novel p3M-2B of 5.9 kb.

p3M-2A promotes the expression of *qnrD* in p3M-2B to enhance the quinolone resistance of host bacteria, in which process *ORF1* plays an important regulatory role, and this regulatory effect becomes more significant with increasing concentration of ciprofloxacin (Figure 2). We speculate here that *ORF1* may code for a small RNA-binding protein and enhance the stability of *qnrD* mRNA by interacting with it, thus improving the expression of *qnrD* and further enhancing the quinolone resistance of P3M. We therefore conjectured that in the long evolutionary process, when the external environment became more and more hostile due to the presence of high concentrations of quinolones, the p3M-2A plasmid played a regulatory role, promoting P3M survival. The specific regulation mechanism remains to be fully elucidated in further studies.

DNA recombination is one of the most important factors that lead to changes of bacterial genetic information, during which DR play an important role [24,25,45]. In this study, we found that the existence of DR is a common feature of these plasmids containing *qnrD*, providing the potential to form a new plasmid when needed and showing that the conserved genes could be transferred and also substituted. This allows the recipient bacteria to obtain resistant genes like *qnrD* from the environment and to get a new resistant gene if the surroundings change. We speculate here that some *qnrD*-carrying plasmids larger than 4.3 kb in size, such as p3M-2B, may primordially have been plasmids of 2683-bp, which obtained new DNA sequence through recombination, which formed new ORFs and eventually formed plasmids of different sizes (Figure 7). The newly acquired sequences of novel plasmids probably derived from excised old plasmids, genomes, or unknown surroundings. Here, we only found three plasmids (p3M-2B, pRS12-78, and pMB18) which contained two different DR-C regions that were in line with the above hypothesis, demonstrating that each type of plasmid has its specific formation process and mode of action and the rules proposed may not be universal. We believe that with more research, more similar *qnrD*-carrying plasmids will be isolated, and our hypothesis will be further confirmed.

Together, three conclusions can be drawn based on our present research. First, the plasmids p3M-2A and p3M-2B function synergistically to improve the quinolone resistance of host strains; in this process, p3M-2A plays an important regulatory role through *ORF1*, promoting the expression of *qnrD*. Thorough studies should help to figure out the specific regulatory mechanisms of *ORF1*. Second, *Proteus* strains may play important roles as disseminators or intermediate hosts during the transmission of *qnrD*-carrying plasmids. Finally, DR regions in *qnrD*-carrying plasmids appear to be among the main elements increasing the possibility of homologous recombination during the formation of plasmids, further promoting the transfer and acquisition of ARGs from the environment [46,47]. Indeed, how repeat sequences are identified by specific targets and how resistance genes are excised and transferred should also be further investigated.

## Figures and Tables

**Table 1:** Strains and plasmids used in this study. P3M, *Proteus vulgaris* strain 3M.

Strains or Plasmids ^a^	Relevant Characteristics	Source or Reference
**Strains**		
P3M	Wild-type strain containing p3M-2A and p3M-2B plasmids	This study
P3M-Δ2A	p3M-2A-deficient strain	This study
P3M-Δ2B	p3M-2B-deficient strain	This study
P3M-Δ2AΔ2B	p3M-2A- and p3M-2B-deficient strain	This study
P3M-Δ2A/2A*	p3M-2A-deficient strain complemented with p3M-2A*	This study
P3M-Δ2B/2B*	p3M-2B-deficient strain complemented with p3M-2B*	This study
*Escherichia coli* DH5α	Competent cell for cloning ^b^	CWBIO Company (CW0808S)
DH5α-2A	DH5α complemented with p3M-2A	This study
DH5α-2B	DH5α complemented with p3M-2B	This study
DH5α-2A2B	DH5α complemented with p3M-2A and p3M-2B	This study
DH5α-2B/2A*	DH5α-2B complemented with p3M-2A*	This study
DH5α-2A/2B*	DH5α-2A complemented with p3M-2B*	This study
*E. coli* S17-1	Mobilizing donor strain with streptomycin resistance	[30]
**Plasmids**		
p3M-2A	2656-bp plasmid isolated from P3M	This study
p3M-2B	5903-bp plasmid carrying *qnrD*, isolated from P3M	This study
p3M-2A*	p3M-2A without *ORF1*	This study
p3M-2B*	p3M-2B without *ORF5*	This study

^a^ The asterisks (*) denote deletion of *ORF1* from p3M-2A or deletion of *ORF5* from p3M-2B. ^b^ The *gyrA* mutation in *E. coli* DH5α does not increase the minimum inhibitory concentration (MIC) for ciprofloxacin.

**Table 2:** Determination of the MIC of ciprofloxacin in different transformants with respect to the parental strains.

Strains	MIC to Ciprofloxacin (mg/L) ^a^
P3M	1
P3M-Δ2A	0.5
P3M-Δ2B	0.125
P3M-Δ2AΔ2B	0.125
*E. coli* DH5α-2A2B	0.125
*E. coli* DH5α-2B	0.06
*E. coli* DH5α-2A	0.03
*E. coli* DH5α	0.03

^a^ All MIC determinations were performed by broth microdilution assays according to CLSI standards.

## Supplementary Materials

The following are available online at https://www.mdpi.com/2076-2607/8/7/1074/s1, Table S1: Primers used in this study, Table S2: *qnrD*-carrying plasmids isolated from different strains.
