# Supplementary material for: Functional Identification and Evolutionary Analysis of Two Novel Plasmids Mediating Quinolone Resistance in Proteus vulgaris
Source: Microorganisms. 2020 Jul 18;8(7):1074. doi: 10.3390/microorganisms8071074 (PMC7409132; doi:10.3390/microorganisms8071074)
Supplement: Supplementary file 1 [file microorganisms-08-01074-s001.zip › microorganisms-870096-SI/Table S1-07152020.pdf]

Table S1. Primers used in this study

Primer [a]	Sequence (5’-3’) [b]	Purpose
2A*-F	GGATCCAAATAGTGCCTCTGGGCAAA	Amplification primers of p3M-2A*
2A*-R	GGATCCACGGGCGAGTTAGTTAGCAA	
2B*-F	GGATCCGTATGAATCAATTTTAGCTAGAG	Amplification primers of p3M-2B*
2B*-R	GGATCCAATCCCTTTTATGTTAAACGG	
q3M-16S-F	TCGCCTAGGTGAGCCTTTAC	Primers of reference gene 16S for qRT-PCR in P3M and corresponding strains
q3M-16S-R	CGAGCGGTAACAGGAGAAAG	
qDH-16S-F	CGGACGGGTGAGTAATGTCT	Primers of reference gene 16S for qRT-PCR in DH5α and corresponding strains
qDH-16S-R	TCAGACCAGCTAGGGATCGT	
q-*qnrD*-F	GCTCAAGGAGCTGATTTTCG	Primers of *qnrD* for qRT-PCR
q-*qnrD*-R	ACGATGAAAACTCCCCACAA	
YZ-2A-F	GGTTTGCATCGCATTTTACA	Validation primers of p3M-2A and p3M-2A*
YZ-2A-R	AGCTTTCGACACCTCCAAGA	
YZ-2B-F	CAGTTGCCCAGCTATGTTGA	Validation primers of p3M-2B and p3M-2B*
YZ-2B-R	TCTGTCACTGCATCCAAACC	

[a] F, forward primer; R, reverse primer.

[b] Restriction sites are underlined.
