# Supplementary material for: Functional Identification and Evolutionary Analysis of Two Novel Plasmids Mediating Quinolone Resistance in Proteus vulgaris
Source: Microorganisms. 2020 Jul 18;8(7):1074. doi: 10.3390/microorganisms8071074 (PMC7409132; doi:10.3390/microorganisms8071074)
Supplement: Supplementary file 1 [file microorganisms-08-01074-s001.zip › microorganisms-870096-SI/Table S2-07152020.pdf]

Table S2. *qnrD*-carrying plasmids isolated from different strains.

Plasmids[a]	Sizes (bp)	Strains	Sources[b]	Accession numbers
pBT169	2683	*Providencia alcalifaciens* strain BT169	Coastal water	MH085194
pAB213	2683	*Providencia rettgeri* strain AB213	Lettuce	MH085193
pOA8911	2683	*Ochrobactrum anthropi*	--	HF913789
pOA8917	2683	*Ochrobactrum anthropi*	--	HF913788
pOA8912	2683	*Ochrobactrum anthropi*	--	HF679278
p36852	2683	*Proteus vulgaris* strain 36852	Bovine	MF062093
p22499	2683	*Proteus penneri* strain 22499	Goat	MF062092
p39190	2669	*Proteus mirabilis* strain 39190	Dog	MF062091
p36854	2669	*Proteus mirabilis* strain 36854	Bovine	MF062090
p33184	2657	*Proteus mirabilis* strain 33184	Dog	MF062089
p2007057	4270	*Salmonella enterica subsp. enterica serovar Bovismorbificans*	Human infections	FJ228229
pSE10MM	2662	*Morganella morganii* strain SE10MM	Urine clinical sample	KU160530
KVHS-001	6887	*Salmonella enterica subsp. enterica serovar Braenderup* strain 95.50	--	KJ685894
KVHS-002	8127	*Salmonella enterica subsp. enterica serovar Typhimurium* strain 14620	--	KJ685893
KVHS-003	9384	*Salmonella enterica subsp. enterica serovar Montevideo* strain 29722/48	--	KJ685891

KVHS-004	6227	*Salmonella enterica subsp. enterica serovar Hadar* strain 139.67	--	KJ685892
pPmZXF	2683	*Proteus mirabilis* strain PmZXF	Chicken	KP313759
p1042	2682	*Proteus mirabilis* strain C1042	Chicken	KP330456
pMB18	5201	*Proteus vulgaris* strain MB18	Urine	KM577619
pEAD1-2	2669	*Proteus mirabilis* strain Q1084	Urine	KF498971
pEAD1-1	2683	*Proteus vulgaris* strain Q5169	Surgery wound	KF498970
pM510	2683	*Proteus mirabilis*	Housefly	KJ190020
pLRB12-304	2658	*Proteus mirabilis*	--	KF364957
pRS12-189	2656	*Proteus mirabilis*	--	KF364956
pRS12-104	2683	*Proteus mirabilis*	--	KF364955
pRS12-78	4286	*Proteus vulgaris*	--	KF364954
pRS12-11	2683	*Proteus mirabilis*	--	KF364953
p3M-2B	5903	*Proteus vulgaris* strain 3M	Shrimp	JX514066
pCGH69	2683	*Morganella morganii* strain CGH69	Urine	JQ776510
pCGP248	2683	*Proteus mirabilis* strain CGP248	Dog feces	JQ776503
pCGF41	4268	*Citrobacter freundii* strain CGF41	Pork	JQ776505
pCGB40	4269	*Escherichia coli* strain CGB40	Pigeon feces	JQ776504
p831	2684	*Morganella morganii* strain Vr831	Urine	JN183061
pT80	2687	*Proteus mirabilis* strain T80	Urine	JN183060
pGHS09-09a	2683	*Providencia rettgeri*	--	HQ834473
pDIJ09-518a	2683	*Providencia rettgeri*	--	HQ834472
pCGP246	4270	*Escherichia coli* strain CGP246	Dog feces	JQ776501
pCGP169	4270	*Escherichia coli* strain CGP169	Dog feces	JQ776502
pCGH25	4270	*Klebsiella pneumoniae* strain	Urine	JQ776509

		CGH25		
pCGP180	4270	*Proteus mirabilis* strain CGP180	Canis lupus familiaris feces	JX982605
pCGH40	4270	*Proteus mirabilis* strain CGH40	Urine	JX982606
p39224	2683	*Proteus mirabilis* strain 39224	Dog	MF062094
pIB_COL3M	2655	*Proteus mirabilis* strain CRE14IB	Urine catheter	NZ_CP045539
pOA8916	1961	*Ochrobactrum anthropi*	--	HF913787
pQnrD2	4268	*Salmonella enterica subsp. enterica serovar Hadar* strain N08-2312	Homo sapiens feces	KJ158441
Unnamed	2683	*Salmonella enterica subsp. enterica serovar Heidelberg* strain 69	Swine	MK191843
pM60	2683	*Morganella morganii*	Urine	KF813021

[a] The sequences in the GenBank entries for KVHS-001, KVHS-002, KVHS-003 and KVHS-004 are linear and these entries do not state that they are complete plasmids, which appear to be fragments.

[b] "--" means the source remains unknown or unreported.
